# Is Long-term mental health care in Portugal ensuring psychosocial rehabilitation? – analysis of the discharge destinations

**DOI:** 10.1192/j.eurpsy.2025.803

**Published:** 2025-08-26

**Authors:** F. Santos Martins, R. Malta, J. V. Santos

**Affiliations:** 1 Psychiatry, ‘S. João’ Local Health Unit (ULS S. João); 2 CINTESIS; 3 Neurosciences and Mental Health; 4 MEDCIDS, Faculty of Medicine, University of Porto (FMUP); 5 Public Health Unit, ‘Santo António’ Local Health Unit, Porto, Portugal

## Abstract

**Introduction:**

Since 2017, the reform of mental health services implemented in Portugal has included the establishment of a national network of long-term mental health care (MH-LTC) to promote the psychosocial rehabilitation of people with mental illness. Different facilities are available according to the degree of psychosocial disability, functionality and family and social support network. Thus, residential structures, home support teams, and socio-occupational units are available to reintegrate these users into society and their families.

**Objectives:**

To assess discharge destinations according to the MH-LTC typology (home care teams, residential structures and socio-occupational units).

**Methods:**

We conducted a national retrospective observational study to analyse the MH-LTC discharge destinations using secondary data publicly available. The following discharge destination categories were considered in the analysis: home (with or without support), social facilities, nursing home, other typologies from the long-term care network (LTC), and others. The analysis included the discharge destinations between May 2018 and March 2024.

**Results:**

A total of 119 discharges were recorded, with 50 patients (42.0 %) going home, four (3.3%) to social facilities, 46 (38.7%) to other LTC typologies and 19 (16.0%) to unspecified destinations. Regarding discharge to home, 27 (54.0%) came from home care teams, 14 (28.0%) from socio-occupational units and 9 (18.0%) from residential facilities. The remaining discharge destinations included 34 (49.3%) from home care teams, 13 (18.8%) from socio-occupational units and 22 (31.9%) from residential facilities.

**Conclusions:**

Although these results do not allow us to gauge the level of disability before and after joining the MH-LTC, they raise some questions. Firstly, the number of discharges is small considering the span of more than five years. In addition, non-residential facilities have the highest number of discharges compared to residential facilities. On the other hand, less than half of the discharge destinations are to the patient’s homes, which may lead us to question whether the MH-LTC fulfils its purpose of psychosocial rehabilitation or whether it is a transitional structure aimed at responding to social issues.

**Disclosure of Interest:**

None Declared

